# MolPROP: Molecular Property prediction with multimodal language and graph fusion

**DOI:** 10.1186/s13321-024-00846-9

**Published:** 2024-05-22

**Authors:** Zachary A. Rollins, Alan C. Cheng, Essam Metwally

**Affiliations:** Modeling and Informatics, Merck & Co., Inc., South San Francisco, CA USA

**Keywords:** Molecular properties, Graph, Language, Multimodal, Deep Learning

## Abstract

**Abstract:**

Pretrained deep learning models self-supervised on large datasets of language, image, and graph representations are often fine-tuned on downstream tasks and have demonstrated remarkable adaptability in a variety of applications including chatbots, autonomous driving, and protein folding. Additional research aims to improve performance on downstream tasks by fusing high dimensional data representations across multiple modalities. In this work, we explore a novel fusion of a pretrained language model, ChemBERTa-2, with graph neural networks for the task of molecular property prediction. We benchmark the MolPROP suite of models on seven scaffold split MoleculeNet datasets and compare with state-of-the-art architectures. We find that (1) multimodal property prediction for small molecules can match or significantly outperform modern architectures on hydration free energy (FreeSolv), experimental water solubility (ESOL), lipophilicity (Lipo), and clinical toxicity tasks (ClinTox), (2) the MolPROP multimodal fusion is predominantly beneficial on regression tasks, (3) the ChemBERTa-2 masked language model pretraining task (MLM) outperformed multitask regression pretraining task (MTR) when fused with graph neural networks for multimodal property prediction, and (4) despite improvements from multimodal fusion on regression tasks MolPROP significantly underperforms on some classification tasks. MolPROP has been made available at https://github.com/merck/MolPROP.

**Scientific contribution:**

This work explores a novel multimodal fusion of learned language and graph representations of small molecules for the supervised task of molecular property prediction. The MolPROP suite of models demonstrates that language and graph fusion can significantly outperform modern architectures on several regression prediction tasks and also provides the opportunity to explore alternative fusion strategies on classification tasks for multimodal molecular property prediction.

## Introduction

Learned molecular representations have undergone rapid evolution in recent years exploring a variety of encoding mechanisms including string line annotations (e.g., SMILES [1], SMARTS [2], or SELFIES [3]) and graph representations [4]. These representations are commonly pretrained in a self-supervised fashion and/or supervised on downstream tasks such as molecular property prediction. While line annotations such as SMILES (Simplified Molecular-Input Line Entry System) [1] strings are compact, have a well-defined grammar, and contain large accessible datasets for self-supervised pretraining, language models do not explicitly encode physical information about molecular topology. This has prompted efforts to represent molecules as graphs to explicitly capture connectivity information. Indeed, significant progress has been demonstrated in terms of sample efficiency and generalizability to new molecules by explicitly representing molecules as graph neural networks (GNNs) where local information is aggregated and passed (i.e., message passing) across the graph structure according to its connectivity [5–11]. Additional work has also explored incorporating 3D information such as bond distances [12, 13] or pretraining GNNs on various tasks such as atom masking or subgraph removal [14].

**Fig. 1:**
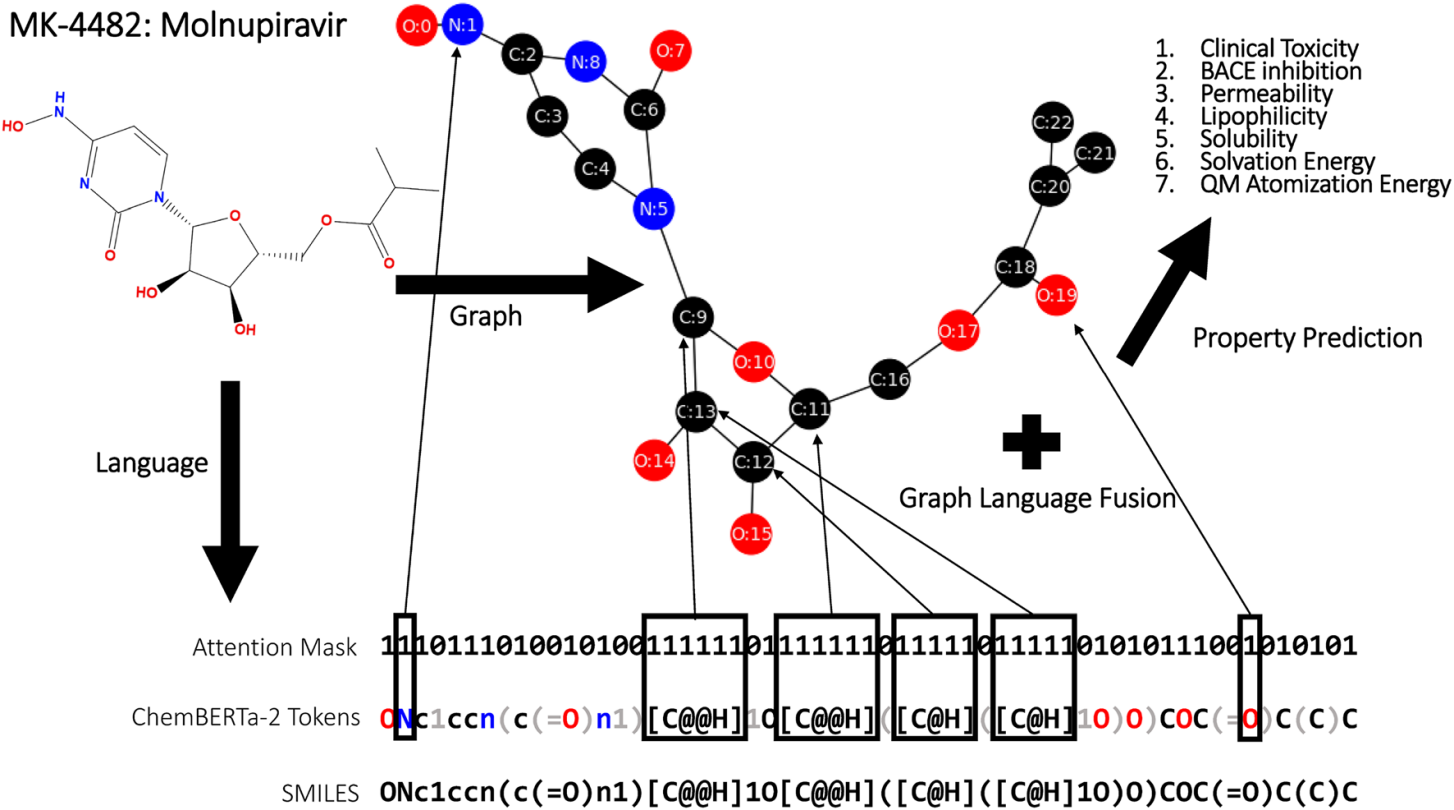
Graphic of the MolPROP architecture. This includes an example of the molecule Molnupiravir. The molecule (top left) is represented as a heavy atom graph (e.g., C, N, O) with nodes defined as circles and edges as lines connecting the circles. The molecule is also represented as a SMILES string (bottom). The ChemBERTa-2 tokenized language representation is shown above the SMILES string where each token is defined by a color change (e.g., [C@@H] is one token). The attention mask is displayed above the token representation which assigns (1) or does not assign (0) attention to the token within the ChemBERTa-2 transformer during fine-tuning of the MolPROP models. The color scheme is defined as carbon=black, nitrogen=blue, oxygen=red, and gray=tokens not assigned attention (0) during fine-tuning and graph fusion. The small black arrows and boxes depict the token representations being concatenated onto their respective graph node features during language and graph fusion

While identifying the best pretraining task for language models and GNNs continues to be an active area of research, language models have demonstrated evidence of the scaling hypothesis across multiple domains including natural language [15], protein language [16–18], and molecular language [19–22]. The scaling hypothesis states that model representational power will continue to grow with increased compute, model size, and pretraining data [23]. In this work, we aim to leverage the representational power of a pretrained language model, ChemBERTa-2 [20], by fusing the language representation to graph representations during fine-tuning on the task of molecular property prediction. We explore ChemBERTa-2 language models [20] pretrained on 77 million molecules from PubChem [24] for two separate tasks: masked language modeling (MLM) and multitask regression (MTR). The ChemBERTa-2 SMILES language model contains a maximum vocabulary size of 591 tokens and maximum context length of 512 tokens. The MLM model, ChemBERTa-2-77 M-MLM, is pretrained to predict randomly masked tokens (15%) in the input SMILES string and the MTR model, ChemBERTa-2-77 M-MTR, is pretrained to predict 200 normalized molecular properties from RDKit [25]. In this work, the language models are fused to the molecular graph representations by mapping the heavy atom tokens to the corresponding heavy atom nodes in the graph (Fig. 1). We explore the fusion of these SMILES language models with the graph convolutional network (GCN) [5] and graph attention network (GATv2) [7] architectures.

The MolPROP suite of models aims to investigate language and graph synergy for the task of molecular property prediction. The MolPROP training objectives span classification and regression tasks from quantum mechanical properties of molecules such as atomization energy to qualitative physiological outcomes such as clinical toxicity (Fig. 1). We find that predominantly regression tasks benefit from the fusion of language and graph representations while the benefit is less clear for classification tasks. The demonstrated synergy on regression tasks suggests that language and graph integration can be beneficial for numerous prediction tasks and also provides opportunities to explore alternative fusion strategies for multimodal molecular property prediction.

## Methods

### Datasets

We evaluate the MolPROP suite of models on 4 regression and 3 classification tasks from the MoleculeNet [26] datasets which range in size (100 s to 1000 s of examples). The regression task datasets include hydration free energy (FreeSolv), experimental water solubility (ESOL), lipophilicity (Lipo), and quantum mechanical atomization energy (QM7). The classification task datasets include inhibitory binding of human $\beta$ secretase (BACE), blood brain barrier penetration (BBBP), and clinical toxicity (ClinTox). The datasets are split into training, validation, and test sets (80/10/10) according to the Bemis-Murko scaffold split procedure from DeepChem [27] designed to be challenging, realistic, and comparable to other published models. Ensembles of models are trained by randomly 10-fold splitting the training/validation set to estimate uncertainty on the test set. Models were first categorized into supervised, supervised with graph pretraining, supervised with language pretraining, and supervised with language and graph. The significance of difference between models is computed from the mean and standard deviation with a two-tailed t-test and the p-values represent the confidence level of the significance test (i.e., * = 95% confidence or p < 0.05, ** = 99% confidence or p < 0.01, and ***= 99.9% confidence p < 0.001). The best performing models from each category were compared throughout the manuscript.

### Language and graph model fusion

ChemBERTa-2 Language Model: ChemBERTa-2 is a BERT-like [28] language model with $\sim$46 M parameters adapted from the RoBERTa [29] architecture. The model is pretrained on a large corpus of 77 million SMILES strings aggregated from PubChem [24]. The 591 length token vocabulary was annotated from common SMILES characters found in the PubChem [24] dataset and a maximum sequence length of 512 tokens was set during the pretraining phase. Although masked language modeling is the most common pretraining task for language models, there is at least a hypothesis that this pretraining task may be insufficient due to an overrepresentation of carbon tokens in small molecule datasets. Therefore, we include and explore two pretraining tasks for fusing ChemBERTa-2 language representations: masked language modeling (MLM) and multitask regression (MTR). The MLM task, ChemBERTa-2-77 M-MLM, is a standard masked language modeling task where 15% of the tokens are randomly masked, and the model is trained to predict the masked tokens. The MTR task, ChemBERTa-2-77 M-MTR, is trained to predict 200 mean-normalized molecular properties calculated in RDKit [25] directly from SMILES strings.

Graph Neural Networks: Graphs are seamless representations of molecules commonly defined by nodes as atoms (n) and edges as bonds (e) of a given graph structure G(n,e). In graph neural networks (GNNs), the nodes update their state by aggregating information from the edges of neighboring nodes. GNN architectures differ in the mechanism by which information is aggregated and combined to update the node states. In this work, we explore two GNN architectures: graph convolutional networks (GCN) [5] and graph attention networks (GATv2) [7]. The GCN architecture is a common message passing neural network where the spectral graph convolution is defined by estimating the product of the graph nodes and a diagonalized filter using a first order approximation of Chebyshev polynomials [5]. The GATv2 architecture is a recent extension of the original transformer generalization on graphs (GAT) [6]. In short, this attention-based neighborhood aggregation mechanism updates node states by computing a weighted average of its attended neighbors [7]. We elected to explore both GCN and GATv2 architectures because performance has been demonstrated to vary between modern GNN architectures depending on the property prediction task. The graphs were constructed using RDKit from SMILES strings and converted to torch geometric objects [30]. The graphs were initialized with node features (atomic number, formal charge, hybridization, and chirality) and edges features (bond type and bond direction). Hydrogens were excluded from the graph representations for the practical purpose of fusing the ChemBERTa-2 language model tokens to the atom node features.

Multimodal fusion: The fusion of language and graph representation is commonly performed in deep learning for proteins because the number of nodes, typically defined as the alpha carbons in the residues, corresponds to the number of residues in the protein. The residue or token embeddings can then be concatenated onto the graph node features during fine-tuning and this generally boosts performance on downstream tasks [31, 32]. In contrast, mapping the tokens from small molecule SMILES string language models to the graph representation is non-trivial because there does not exist a 1:1 mapping of tokens to nodes. For example, in ChemBERTa-2 there are 591 tokens in the vocabulary, but only a subset of those tokens contain unique atom types (H, C, N, O, etc.). Moreover, some tokens contain multiple atoms (e.g., [*C*@@*H*], [$NH2+$], etc.) making the mapping of tokens to nodes ambiguous. In this work, we circumvent this ambiguity by simply extracting the tokens containing heavy atoms and mapping them to the nodes containing heavy atoms (Fig. 1). This is accomplished by assigning attention weight to the heavy atom tokens in the attention mask during fine-tuning and concatenating these heavy atom token representations onto the heavy atom nodes in the small molecule graph representation (i.e., hydrogens are ignored). This is a simple and effective strategy for exploring the fusion of language and graph representations for small molecules, however, future work may explore strategies that include hydrogens and/or a dynamic mapping between the token and graph representations. Moreover, alternative strategies for graph and language fusion may utilize graph pretraining [11, 14], an attention mechanism [33], or convolutional feature extraction of the language representation before concatenating to the graph nodes [34].

**Table 1:** The searched MolPROP hyperparameters spaces

Hyperparameters	MolPROP
lr coder	loguniform(1e-5, 1e-2)
lr lang	loguniform(1e-9, 1e-6)
lr step	randint(1,5)
language freeze layer count	randint(0,3)
dense layer dropout	uniform(0.1, 0.5)

For each model, the respective hyperparameter sets used in 50 sample Bayesian optimization with hyperband (BOHB) runs

### Hyperparameter optimization

Hyperparameters were selected using the Bayesian optimization with hyperband (BOHB) [35] algorithm implemented by Ray Tune [36] (Table 1). This algorithm reduces hyperparameter search wall time up to 50X by combining the sample efficiency of Bayesian optimization and the adaptive sampling/ early stopping advantages of bandit methodologies. Hyperparameters included the learning rates for the language and graph module, the number of layers to freeze in the ChemBERTa-2 language model (0-3), the number of steps for linear increase warm-up, and the dropout fraction in the dense layer (Table 1). The training loss was computed corresponding to previously used metrics in the literature (i.e., RMSE: FreeSolv, ESOL, Lipo; MAE: QM7; BCE: BACE-1, BBBP, ClinTox) where RMSE, MAE, and BCE correspond to the root-mean-square error, mean absolute error, and binary cross entropy, respectively. The best hyperparameter set was selected based on the best performance across the k-fold validation sets after a 50 sample BOHB run (i.e., lowest RMSE: FreeSolv, ESOL, Lipo; lowest MAE: QM7; highest ROC-AUC: BACE, BBBP, ClinTox) where ROC-AUC corresponds to the receiver operating characteristic area under the curve. Final performance is evaluated based on the average and standard deviation of the k models on the test set. The learning rate decay strategy was adopted from BERT with linear increase warm-up and inverse square root decay [28]. All runs were performed for 50 epochs, with batch size 16, at 32-bit precision, utilizing the Adam optimizer [37], and on V100 GPUs.

## Results and discussion

The MolPROP suite of models includes two pretrained ChemBERTa-2 language models (MLM and MTR) [20] fused to two graph neural network architectures (GCN and GATv2) [5, 7] for a total of four models. MolPROP models were benchmarked on seven MoleculeNet datasets [26] that are 80/10/10 split into training, validation, and test sets based on the Bemis-Murko scaffold split implementation from DeepChem [27]. The splits and training losses were defined according to their respective metrics in the literature to allow for fair comparison to modern architectures. All models are hyperparameter optimized using the BOHB algorithm [35] and trained for 50 epochs. We evaluate the performance of MolPROP models on the test set by reporting the average and standard deviation of the k models (k=10) trained on the training/validation set. We find that MolPROP models significantly outperform modern architectures such as Chemprop [9] and MolCLR [14] on hydration free energy (FreeSolv), experimental water solubility (ESOL), and clinical toxicity (ClinTox) tasks. We also find that MolPROP models match modern architectures on lipophilicity (Lipo). However, MolPROP models significantly underperform on quantum mechanical atomization energy (QM7), inhibitory binding of human $\beta$ secretase (BACE), and blood brain barrier penetration (BBBP).

### Baselines

The baseline models for comparison were aggregated from the reported literature and categorized into supervised, supervised with graph pretraining, and supervised with language pretraining. For fair comparison, we only included models that utilized identical performance metrics and Bemis-Murko scaffold splits [27] on the datasets. The supervised models include shallow learning with random forest (RF) [14] and support vector machine (SVM) [14] on molecular fingerprints from RDKit [25]. Additional supervised models include heavy-atom graph neural networks: graph convolution network (GCN) [5], graph attention netowrk (GATv2) [7], graph isomorphism network (GIN) [38], SchNet [12], 3D Infomax [13], MGCN [8], and D-MPNN (Chemprop) [9]. The supervised with graph pretraining include Hu et al. [10], N-Gram [11], MolCLR_GCN_, and MolCLR_GIN_ [14]. Finally, the supervised with language pretraining included ChemBERTa-2-77 M-MLM and ChemBERTa-2-77 M-MTR [20]. If reported, the estimation of uncertainty is included.

**Table 2:** MolPROP and baseline model performance on regression tasks

MODEL	FreeSolv	ESOL	Lipo	QM7
$\#$ Molecules	642	1128	4200	6830
Metric	RMSE	RMSE	RMSE	MAE
Supervised				
RF[14]	*2.03* ± *0.22*	1.07 ± 0.19	0.88 ± 0.04	122.7 ± 4.2
SVM[14]	3.14 ± 0.00	1.50 ± 0.00	0.82 ± 0.00	156.9 ± 0.0
GCN [5]	2.87 ± 0.14	1.43 ± 0.05	0.85 ± 0.08	122.9 ± 2.2
GATv2[7]	3.14 ± 0.00	1.41 ± 0.00	0.89 ± 0.00	113.3 ± 0.0
GIN[38]	2.76 ± 0.18	1.45 ± 0.02	0.85 ± 0.07	124.8 ± 0.7
SchNet[12]	3.22 ± 0.76	1.05 ± 0.06	0.91 ± 0.10	***74.2*** ± ***6.0*** ^*^
3D Infomax[13]	2.23 ± 0.26	*0.947* ± *0.04*	0.739 ± 0.01	—
MGCN[8]	3.35 ± 0.01	1.27 ± 0.15	1.11 ± 0.04	77.6 ± 4.7
D-MPNN (Chemprop)[9]	2.18 ± 0.91	0.98 ± 0.26	***0.65*** ± ***0.05*** ^ns^	105.8 ± 13.2
Supervised with graph pretraining				
Hu et al. [10]	2.83 ± 0.12	1.22 ± 0.02	0.74 ± 0.00	110.2 ± 6.4
N-Gram[11]	2.51 ± 0.19	*1.10* ± *0.03*	0.88 ± 0.12	125.6 ± 1.5
MolCLR_GCN_[14]	2.39 ± 0.14	1.16 ± 0.00	0.78 ± 0.01	*83.1 ± 4.0*
MolCLR_GIN_[14]	*2.20* ± *0.20*	1.11 ± 0.01	***0.65*** ± ***0.08***^ns^	87.2 ± 2.0
Supervised with language pretraining				
ChemBERTa-2-77M-MTR[20]	2.515 ± 0.00	1.025 ± 0.00	0.987 ± 0.00	***147.9*** ± ***0.00***
ChemBERTa-2-77M-MLM[20]	*2.047*± *0.00*	*0.889 ± 0.00*	***0.798*** ± ***0.00***	172.8 ± 0.00
Supervised with Graph and Language				
MolPROP_GCN-ChemBERTa-2-77M-MTR_	2.15 ± 0.14	0.990 ± 0.09	0.812 ± 0.02	163.0 ± 29.8
MolPROP_GATv2-ChemBERTa-2-77M-MTR_	2.05 ± 0.16	0.991 ± 0.11	0.799 ± 0.01	***131.8*** ± ***11.2***
MolPROP_GCN-ChemBERTa-2-77M-MLM_	1.73 ± 0.14	0.806 ± 0.03	0.790 ± 0.02	136.4 ± 19.8
MolPROP_GATv2-ChemBERTa-2-77M-MLM_	***1.70*** ± ***0.09*** ^**^	***0.777*** ± 0.02^***^	***0.733*** ± ***0.02***	151.8 ± 10.0

Model performance is assessed by the metric provided in the header: RMSE = root-mean-square error, MAE = mean absolute error

MolPROP mean and standard deviation of k-models are reported on the test set from 10-fold cross-validation

The baseline performances are reported from the literature. The columns are the model type, performance on the FreeSolv dataset, performance on the ESOL dataset, performance on the Lipo dataset, and performance on the QM7 dataset, respectively

The rows consist of model types separated by categorization: supervised, supervised with graph pretraining, supervised with language pretraining, and supervised with language and graph, respectively

The model category is partitioned by a black horizontal line. The best performing model for each class is *italicized* and the best deep learning model across categories is also bolded

Significance is determined by comparing the best models from their respective category. The significance of difference between means and standard deviations is determined by a two-tailed t-test and the p-values represent the confidence level of the significance test [ns] not statistically significant (p > 0.05)

*statistically significant (p < 0.05)

**statistically significant (p < 0.01)

***statistically significant (p < 0.001)

### MolPROP regression benchmarks

The experimental hydration free energy (FreeSolv) task is a regression task that aims to predict the measured free energy of hydration for a given molecule in kcal/mol. The MolPROP models significantly outperforms baseline models achieving a RMSE 1.70 ± 0.09 and 1.73 ± 0.14 for the MolPROP_GATv2-ChemBERTa-2-MLM_ and MolPROP_GCN-ChemBERTa-2-MLM_ variants, respectively (Table 2). These MolPROP models significantly outperform the best supervised random forest (p=0.002). In addition, the MolPROP models also outperform the best supervised with graph pretraining model MolCLR_GIN_ (p = 0.0001). Importantly, the MolPROP models outperformed fine-tuned versions of the graph-only (GCN and GATv2) and language-only (ChemBERTa-2-77 M-MLM and ChemBERTa-2-77 M-MTR) models in 4/4 cases for the FreeSolv regression task. Next, the experimental water solubility (ESOL) task is a regression task that aims to predict the logarithm of water solubility in mol/L for a given molecule. The MolPROP models significantly outperform the baseline models achieving a RMSE 0.77 ± 0.02. Similarly, the MolPROP models significantly outperform the best supervised 3D Infomax model (p=0.0001). Interestingly, the MolPROP variant MolPROP_GCN-ChemBERTa-2-MLM_ is able to outperform both the GCN graph-only (p=0.0001)and ChemBERTa-2-MLM language-only models demonstrating synergy from representation fusion in the experimental water solubility task. Moreover, the MolPROP synergy is demonstrated by outperforming fine-tuned versions of the graph-only (GCN and GATv2) and language-only (ChemBERTa-2-77 M-MLM and ChemBERTa-2-77 M-MTR) models in 4/4 cases for the ESOL regression task. Next, the lipophilicity (Lipo) task is a regression task that aims to predict the experimental octanol/water distribution coefficient for a given molecule (logP). MolPROP_GATv2-ChemBERTa-2-MLM_ achieves comparable performance on the lipophilicty task to the best baseline models achieving a RMSE 0.733 ± 0.02. Despite competitive MolPROP performance to other baselines, Chemprop and MolCLR_GIN_ significantly outperform on the Lipo regression task. However, similar to the FreeSolv and ESOL tasks, MolPROP models achieved better performance than fine-tuned versions of the graph-only (GCN and GATv2) and language-only (ChemBERTa-2-77 M-MLM and ChemBERTa-2-77 M-MTR) models in 4/4 cases on the Lipo regression task demonstrating synergy from representation fusion. Finally, the quantum mechanical atomization energy (QM7) task is a regression task that aims to predict the total atomization energy for a given molecule. Interestingly, we found that SchNet was the best performing baseline model for quantum mechanical properties despite its underperformance on other tasks (Table 2). MolPROP models significantly underperform SchNet (p<0.001) and other baseline models on the QM7 dataset which is likely because SchNet is a neural network designed to explicitly include paired atomic distances [12]. MolPROP and other baselines explicitly exclude hydrogens and other geometric features (e.g., paired atomic distances) in the graph representation which are critical to learn atomization energy. Future work may explore the fusion of language and graph representations that include hydrogens or additional geometric features to improve performance on quantum mechanical properties. We also found that for MolPROP multimodal representation fusion with ChemBERTa-2-MLM outperformed the ChemBERTa-2-MTR model on 3/4 regression tasks suggesting that the MLM pretraining task is more beneficial for downstream tasks. Overall, we find that MolPROP models can significantly outperform or match modern architectures on experimental water solubility, hydration free energy, and lipophilicity tasks. Moreover, there is synergy from representation fusion on these regression tasks as demonstrated by the improved performance over their language and/or graph only counterparts (Table 2).

**Table 3:** MolPROP and baseline model performance on classification tasks

MODEL	BACE	BBBP	ClinTox
$\#$ Molecules	1513	2039	1478
Metric	ROC-AUC	ROC-AUC	ROC-AUC
Supervised			
RF[14]	*86.7 ± 0.8*	71.4 ± 0.0	71.3 ± 5.6
SVM[14]	86.2 ± 0.0	72.9 ± 0.0	66.9 ± 9.2
GCN[5]	71.6 ± 2.0	71.8 ± 0.9	62.5 ± 2.8
GATv2[7]	57.9 ± 0.0	58.0 ± 0.0	54.1 ± 0.0
GIN[38]	70.1 ± 5.4	65.8 ± 4.5	58.0 ± 4.4
SchNet[12]	76.6 ± 1.1	84.8 ± 2.2	71.5 ± 3.7
3D Infomax[13]	78.1 ± 1.3	68.3 ± 2.0	59.0 ± 5.4
MGCN[8]	73.4 ± 3.0	***85.0 ± 6.4***^ns^	63.4 ± 4.2
D-MPNN (Chemprop)[9]	85.3 ± 5.3	71.2 ± 3.8	*90.5 ± 5.3*
Supervised with graph pretraining			
Hu et al.[10]	85.9 ± 0.8	70.8 ± 1.5	78.9 ± 2.4
N-Gram[11]	87.6 ± 3.5	***91.2 ± 3.0***^ns^	85.5 ± 3.7
MolCLR_GCN_[14]	78.8 ± 0.5	73.8 ± 0.2	86.7 ± 1.0
MolCLR_GIN_[14]	89.0 ± 0.30^**^	73.6 ± 0.5	***93.2 ± 1.7***^ns^
Supervised with language pretraining			
ChemBERTa-2-77M-MTR[20]	73.5 ± 0.0	69.8 ± 0.0	23.9 ± 0.0
ChemBERTa-2-77M-MLM[20]	*79.9 ± 0.0*	*72.8 ± 0.0*	56.3 ± 0.0
Supervised with language and graph			
MolPROP_GCN-ChemBERTa-2-77M-MTR_	68.4 ± 1.8	65.4 ± 1.7	91.0 ± 6.8
MolPROP_GATv2-ChemBERTa-2-77M-MTR_	*68.7 ± 2.0*	63.1 ± 2.3	93.3 ± 3.5
MolPROP_GCN-ChemBERTa-2-77M-MLM_	66.5 ± 3.4	66.0 ± 2.4	94.1 ± 5.1
MolPROP_GATv2-ChemBERTa-2-77M-MLM_	65.6 ± 3.6	*66.3 ± 2.5*	***95.2 ± 3.4***^ns^

Model performance is assessed by the metric provided in the header: ROC-AUC = receiver operating characteristic - area under the curve. MolPROP mean and standard deviation of k-models are reported on the test set from 10-fold cross-validation. The baseline performances are reported from the literature

The columns are the model type, performance on the BACE dataset, performance on the BBBP dataset, and performance on the ClinTox dataset, respectively

The rows consist of model types separated by categorization: supervised, supervised with graph pretraining, supervised with language pretraining, and supervised with language and graph, respectively

The model category is partitioned by a black horizontal line. The best performing model for each class is italicized and the best deep learning model across categories is also bolded. Significance is determined by comparing the best models from their respective category

The significance of difference between means and standard deviations is determined by a two-tailed t-test and the p-values represent the confidence level of the significance test [ns] not statistically significant (p > 0.05)

*statistically significant (p < 0.05)

** statistically significant (p < 0.01)

*** statistically significant (p < 0.001)

### MolPROP classification benchmarks

To extend the MolPROP benchmarks to classification, we evaluated the MolPROP models on three classification tasks from the MoleculeNet datasets: inhibitory binding of human $\beta$ secretase (BACE), blood brain barrier penetration (BBBP), and clinical toxicity (ClinTox). All model performance was assessed by the receiver operating characteristic area under the curve (ROC-AUC). The inhibitory binding of human $\beta$ secretase (BACE) task is a binary classification task that aims to predict whether a given molecule is an inhibitor of BACE. The MolPROP models significantly underperform all baselines on the BACE classification task. The blood brain barrier penetration (BBBP) task is a binary classification task that aims to predict whether a given molecule can penetrate the blood brain barrier (permeability). Similarly, the MolPROP models significantly underperform all baselines on the BBBP classification task (Table 3). Surprisingly, for the BACE and BBBP classification tasks there is no evidence of representation fusion synergy in MolPROP variants demonstrated by the decreased performance compared to the graph-only (GCN and GATv2) and language-only (ChemBERTa-2-MLM and ChemBERTa-2-MTR) models. We further investigate this phenomenon in the "Ablation experiments and embedding visualization" section. Finally, the clinical toxicity (ClinTox) task is a binary classification task that aims to predict whether a given molecule is toxic. Interestingly, the MolPROP models outperforms most baselines on the ClinTox classification task. For example, the MolPROP_GATv2-ChemBERTa-2-MLM_ achieves an ROC-AUC of 95.2 ± 3.4% compared to the best baseline supervised model Chemprop which achieves an ROC-AUC of 90.5 ± 5.3% (p=0.047). Additionally, MolPROP_GATv2-ChemBERTa-2-MLM_ slightly outperforms MolCLR_GIN_ which is the best baseline model with a ROC-AUC 93.2 ± 1.7% (p=0.36). Moreover, all MolPROP variants achieved better performance than their graph-only (GCN and GATv2) and language-only (ChemBERTa-2-MLM and ChemBERTa-2-MTR) counterparts demonstrating synergy from representation fusion on the ClinTox classification task. We also found that multimodal representational fusion with ChemBERTa-2-MLM outperformed ChemBERTa-2-MTR on 2/3 classification tasks suggesting that the MLM pretraining task is more beneficial for downstream tasks (Table 3). Overall, we find that deep learning models can benefit from representational fusion for classification tasks such as clinical toxicity, however, the guidelines for when and how representational fusion is beneficial remains an open question. Recent investigations have found that GNNs can be unstable when training on classifications tasks [39–43]. For example, GCNs have been reportedly unstable when the number of node features becomes too large [42] and GATv2 suffers from initialization instabilities [43]. These fundamental limitations of the GNNs may explain the more variable performance on classification tasks. Future work may explore alternative graph and language fusion strategies for small molecule classification tasks that utilizes graph pretraining [11, 14], an attention mechanism [33], or convolutional feature extraction of the language representation [34]. Additional strategies may include distilling the language model embeddings from ChemBERTa before concatenating to the graph nodes to improve GCN and GATv2 stability on classification tasks.

**Table 4:** MolPROP ablation experiments on regression and classification tasks

MODEL	FreeSolv	ESOL	BACE	BBBP	ClinTox
$\#$ Molecules	642	1128	1513	2039	1478
Metric	RMSE	RMSE	ROC-AUC	ROC-AUC	ROC-AUC
GATv2 (ablated)	2.72 ± 0.14	1.56 ± 0.12	54.6 ± 3.3	51.0 ± 1.5	52.5 ± 1.5
ChemBERTa-2-77M-MLM (ablated)	1.78 ± 0.18	0.81 ± 0.02	68.1 ± 4.9	65.3 ± 4.2	87.2 ± 13.4
MolPROP_GATv2-ChemBERTa-2-77M-MLM_	1.70 ± 0.09	0.777 ± 0.02	65.6 ± 3.6	*66.3* ± *2.5*	95.2 ± 3.4

Model performance is assessed by the metric provided in the header: RMSE = root-mean-square error and ROC-AUC = receiver operating characteristic - area under the curve. MolPROP mean and standard deviation of k-models are reported on the test set from 10-fold cross-validation.The columns are the model type, performance on the FreeSolv dataset, performance on the ESOL dataset, performance on the BACE dataset, performance on the BBBP dataset, and performance on the ClinTox dataset, respectively

### Ablation experiments and embedding visualization

We further investigate the performance of the MolPROP fusion strategy by performing ablation experiments on the MolPROP_GATv2-ChemBERTa-2-77 M-MLM_ architecture for both regression and classification tasks. We elect to perform ablation experiments on the best performing regression tasks, FreeSolv and ESOL, as well as all the classification tasks: BACE, BBBP, and ClinTox. The ablation experiments are performed by utilizing the exact hyperparameters found during hyperparameter optimization in (Table 2, 3) and retraining the models with the GATv2 or ChemBERTa-2-77 M-MLM model ablated from the architecture. For regression tasks, we demonstrate that the ablated GATv2 model underperforms the ablated ChemBERTa-2-77 M-MLM model on both FreeSolv and ESOL tasks; however, the MolPROP_GATv2-ChemBERTa-2-77 M-MLM_ fusion is able to synergistically fuse both molecular representations to outperform either GATv2 or ChemBERTa-2-77 M-MLM alone (Table 4). For classification tasks, we find that the ablated GATv2 model similarly underperforms the ablated ChemBERTa-2-77 M-MLM model on both BACE and BBBP tasks (Table 4). In contrast to the regression tasks, the MolPROP_GATv2-ChemBERTa-2-77 M-MLM_ fusion strategy is not synergistic for these classification tasks and only achieves similar performance to the ChemBERTa-2-77 M-MLM ablated model (Table 4). Despite no synergy on the BACE and BBBP classification tasks, the ClinTox task demonstrates that the MolPROP_GATv2-ChemBERTa-2-77 M-MLM_ fusion strategy is able to synergize molecular representations from the ablated GATv2 and ChemBERTa-2-77 M-MLM models (Table 4). Overall, these results suggest that the MolPROP fusion strategy is predominantly beneficial for regression tasks. Future work may explore alternative fusion strategies to improve the stability of multimodal fusion on classification tasks such as graph pretraining [11, 14], attention mechanisms [33] or convolutional feature extraction of the language representation [34].

**Fig. 2:**
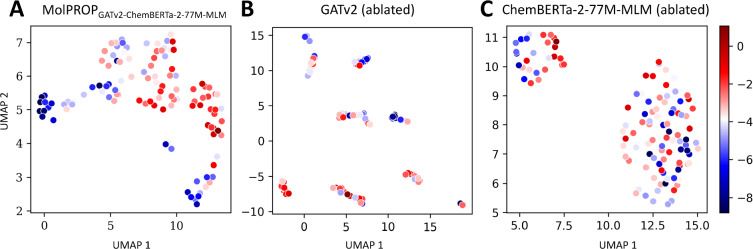
Latent Embedding Visualization of the MolPROP ESOL Regression Model. The learned neural network embeddings of the ESOL test set are projected into 2-dimensional space utilizing the UMAP algorithm for **A** MolPROP_GATv2-ChemBERTa-2-77 M-MLM_, **B** GATv2 (ablated), and **C** ChemBERTa-2-77 M-MLM (ablated) models. All panels display the 1st UMAP dimension as the *x*-axis and the 2nd UMAP dimesion as the *y*-axis. The 2-dimensional UMAP projection is determined with the 10 nearest neighbors, utilizing the Chebyshev distance metric, and a minimum distance of 0.25. The color scheme is displayed on the right panel as a colorbar where the scalar values range from red to blue and represent the logarithm of water solubility in mol/L. Therefore, red clusters of molecules have high water solubility and the blue clusters of molecules have low water solubility

**Fig. 3:**
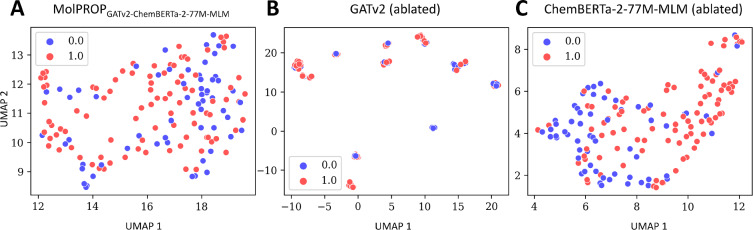
Latent Embedding Visualization of the MolPROP BACE Classification Model. The learned neural network embeddings of the BACE test set are projected into 2-dimensional space utilizing the UMAP algorithm for **A** MolPROP_GATv2-ChemBERTa-2-77 M-MLM_, **B** GATv2 (ablated), and **C** ChemBERTa-2-77 M-MLM (ablated) models. All panels display the 1st UMAP dimension as the *x*-axis and the 2nd UMAP dimesion as the *y*-axis. The 2-dimensional UMAP projection is determined with the 10 nearest neighbors, utilizing the Jaccard distance metric, and a minimum distance of 0.25. The color scheme is displayed in each panel as a binary blue or red circle. The discrete binary values represent the no inhibition (i.e., blue or 0) or inhibition (i.e., red or 1) of human $\beta$ secretase, BACE

**Fig. 4:**
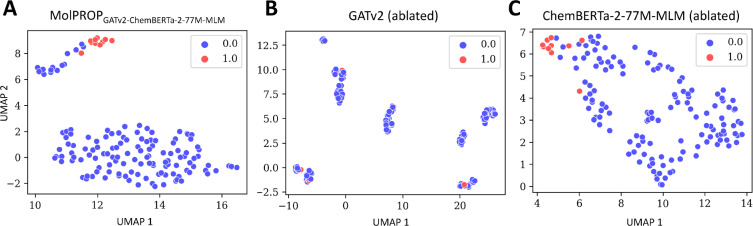
Latent Embedding Visualization of the MolPROP ClinTox Classification Model. The learned neural network embeddings of the ClinTox test set are projected into 2-dimensional space utilizing the UMAP algorithm for **A** MolPROP_GATv2-ChemBERTa-2-77 M-MLM_, **B** GATv2 (ablated), and **C** ChemBERTa-2-77 M-MLM (ablated) models. All panels display the 1st UMAP dimension as the *x*-axis and the 2^nd^ UMAP dimesion as the *y*-axis. The 2-dimensional UMAP projection is determined with the 10 nearest neighbors, utilizing the Jaccard distance metric, and a minimum distance of 0.25. The color scheme is displayed in each panel as a binary blue or red circle. The discrete binary values represent non-toxic (i.e., blue or 0) or toxic (i.e., red or 1) molecules in clincal trials

Finally, we explore the learned latent embeddings representations of the MolPROP_GATv2-ChemBERTa-2-77 M-MLM_ model by projecting the latent embeddings onto a 2D space using UMAP [44] (Fig. 2, 3, 4). For the ESOL regression task, we find that the MolPROP_GATv2-ChemBERTa-2-77 M-MLM_ model (Fig. 2A) is able to learn molecular representations that are well separated in the 2D space as compared to the GATv2 (ablated) (Fig. 2B and ChemBERTa-2-77 M-MLM (ablated) (Fig. 2C) counterparts. The learned embeddings are able to cluster molecules with similar properties such as the red clusters of molecules with high water solubility and the blue cluster of molecules with low water solubility. The increased separation of clusters for MolPROP_GATv2-ChemBERTa-2-77 M-MLM_ compared to the ablated counterparts demonstrates fusion synergy for the ESOL regression task (Fig. 2).

In contrast, for the BACE classification task, we find that the MolPROP_GATv2-ChemBERTa-2-77 M-MLM_ model (Fig. 3A) is unable to learn molecular representations that are well separated in the 2D space for the MolPROP fusion, GATv2 (ablated), or ChemBERTa-2-77 M-MLM (ablated) models (Fig. 3). The learned embeddings are unable to cluster molecules with similar properties demonstrated by substantial overlap of the red clusters of molecules that are BACE inhibitors and the blue clusters of molecules that are not BACE inhibitors. The inability to effectively fuse information on the BACE classification task may be due to the inability of the individal ablated models to learn sufficient molecular representations (Fig. 3B-C). Previous reports demonstrate that graph pretraining is an effective strategy to learn molecular molecular representations (Table3) for the BACE [14] and BBBP [11] classification tasks. Future work may explore graph pretraining strategies to improve the MolPROP fusion strategy for classification tasks.

Similar to the ESOL regression task, the MolPROP_GATv2-ChemBERTa-2-77 M-MLM_ model is able to learn molecular representations that are well separated in the 2D space for the ClinTox classification task (Fig. 4A). The learned embeddings are able to cluster molecules with similar properties such as the red cluster of molecules that are toxic and the blue cluster of molecules that are non-toxic. This learned latent representation demonstrates improved separation of the red and blue clusters in the MolPROP fusion model as compared to the GATv2 (ablated) (Fig. 4B) and ChemBERTa-2-77 M-MLM (ablated) (Fig. 4C) models. Moreover, unlike the BACE and BBBP classification tasks, the MolPROP fusion is able to further improve the learned molecular representation from the ChemBERTa-2-77 M-MLM (ablated) (Fig. 4C) model. This result suggests that the ability for the MolPROP fusion to be effective, there needs to be sufficient molecular representations learned from the individual ablated models.

## Conclusion

We present a novel suite of models for molecular property prediction, MolPROP, utilizing multimodal representation fusion of pretrained language and graph neural networks. We demonstrate that representation fusion can be beneficial for regression and classification tasks such as experimental water solubility, hydration free energy, lipophilicity, and clinical toxicity. However, we also find that representational fusion can underperform on quantum mechanical atomization energy, inhibitory binding of human $\beta$ secretase, and blood brain barrier penetration. In general, this multimodal fusion method performs better on regression tasks. Ablation experiments and latent embeddings visualizations reveal that a sufficient learned representation by the individual models may be necessary in order to achieve performance benefit. Comparison of language model pretraining reveals ChemBERTa-2-MLM pretraining task outperforms the ChemBERTa-2-MTR pretraining when tokens are fused to graph representations. We find that there can be performance benefits from multimodal representational fusion for molecular property predictions, and we expect that these improvements will continue to benefit from future advancements in molecular language models. Moreover, there are additional opportunities to improve the algorithmic fusion of molecular graph and language representations particularly for classification tasks.

## Data Availability

The molecular SMILES strings and corresponding datasets are available https://moleculenet.org/datasets-1 . The training and inference code as well as the model weights & hyperparameters are included in MolPROP: https://github.com/merck/MolPROP.
